# Identification of Two Distinct Classes of the Human INO80 Complex Genome-Wide

**DOI:** 10.1534/g3.117.300504

**Published:** 2018-03-06

**Authors:** John S. Runge, Jesse R. Raab, Terry Magnuson

**Affiliations:** † Curriculum for Genetics and Molecular Biology; ‡ Lineberger Comprehensive Cancer Center; § Department of Genetics

**Keywords:** INO80 Complex, Polycomb Repressive Complex 2, P300, H3K27me3, H3K27ac, Genome Report

## Abstract

Chromatin remodeling and histone modifying enzymes play a critical role in shaping the regulatory output of a cell. Although much is known about these classes of proteins, identifying the mechanisms by which they coordinate gene expression programs remains an exciting topic of investigation. One factor that may contribute to the targeting and activity of chromatin regulators is local chromatin landscape. We leveraged genomic approaches and publically-available datasets to characterize the chromatin landscape at targets of the human INO80 chromatin remodeling complex (INO80-C). Our data revealed two classes of INO80-C targets with distinct chromatin signatures. The predominant INO80-C class was enriched for open chromatin, H3K27ac, and representative subunits from each of the three INO80-C modules (RUVBL1, RUVBL2, MCRS1, YY1). We named this class Canonical INO80. Notably, we identified an unexpected class of INO80-C targets that contained only the INO80 ATPase and harbored a repressive chromatin signature characterized by inaccessible chromatin, H3K27me3, and the methyltransferase EZH2. We named this class Non-Canonical INO80 (NC-INO80). Biochemical approaches indicated that INO80-C and the H3K27 acetyltransferase P300 physically interact, suggesting INO80-C and P300 may jointly coordinate chromatin accessibility at Canonical INO80 sites. No interaction was detected between INO80-C and EZH2, indicating INO80-C and EZH2 may engage in a separate form of regulatory crosstalk at NC-INO80 targets. Our data indicate that INO80-C is more compositionally heterogenous at its genomic targets than anticipated. Moreover, our data suggest there is an important link between INO80-C and histone modifying enzymes that may have consequences in developmental and pathological contexts.

ATP-dependent chromatin remodeling complexes (remodelers) comprise a class of highly conserved protein complexes that mediate nucleosome position throughout the genome (Clapier and Cairns 2009). Each remodeling complex contains a catalytic ATPase that breaks histone-DNA contacts using ATP hydrolysis and often includes additional proteins that facilitate nucleosome targeting and mobilization (Clapier and Cairns 2009; Runge *et al.* 2016). Much is known regarding the composition and molecular capabilities of remodelers, yet the factors regulating the function of remodelers at genomic targets remain elusive. Because remodelers dictate the position of nucleosomes and in turn the regulatory output of epigenomic loci, understanding the factors that contribute to the targeting and local function of remodelers is a key aspect in chromatin regulation.

To approach the question of how the localization and function of remodelers are influenced by local chromatin architecture, we elected to study the human INO80 Complex (INO80-C). Details regarding the structural and biochemical assembly of INO80-C *in vitro* are well understood. Originally identified in a genetic screen for *S. cerevisiae* mutants sensitive to inositol (Ebbert *et al.* 1999), the catalytic ATPase of INO80-C, named INO80, was subsequently purified in a large complex that interacted with chromatin, hydrolyzed ATP, and mobilized nucleosomes in yeast (Shen *et al.* 2000). Crystal and electron microscopy structures of the yeast INO80-C recapitulated its nucleosome-targeting function (Tosi *et al.* 2013; Watanabe *et al.* 2015). Additionally, yeast INO80-C subunits have been shown to stimulate or facilitate nucleosome targeting and ATPase activity *in vitro* (Jónsson *et al.* 2004; Chambers *et al.* 2012; Watanabe *et al.* 2015; Yao *et al.* 2016; Willhoft *et al.* 2016). Loss of these proteins confers sensitivity to metabolic stress, polyploidy, and reduces cellular fitness (Chambers *et al.* 2012; Yao *et al.* 2016; Gowans *et al.* 2018). Interestingly, INO80-C subunits may pre-assemble before integrating into the complex (Yao *et al.* 2016; Zhou *et al.* 2017).

The INO80 protein serves as a scaffold for the rest of the complex in human cells (Chen *et al.* 2011), in agreement with how INO80-C is thought to assemble in yeast (Gerhold and Gasser 2014). Three sets of subunits form distinct modules through interaction with specific regions of the human INO80 protein (Chen *et al.* 2011) (Figure 1A). There are 15 mammalian constituents in total that comprise INO80-C (Jin *et al.* 2005; Chen *et al.* 2011). Two modules (Module 1 and Module 2, Figure 1A) are critical for INO80 hydrolytic and remodeling activities, where mutants lacking the modules render INO80-C unable to hydrolyze ATP, and recognize or slide nucleosomes *in vitro* (Cai *et al.* 2007; Chen *et al.* 2011, 2013). A third module (Module 3) is considered dispensable for these functions *in vitro* but contains DNA-binding and protein-modifying proteins potentiating an auxiliary function (Yao *et al.* 2008; Chen *et al.* 2011, 2013). These data suggest that the INO80 ATPase is more than the molecular engine for INO80-C. Instead, INO80 likely serves as a docking site for its subunits in a manner that is critical to its identification of suitable genomic targets and local remodeling function. We hypothesized that INO80 responds to site-specific chromatin-based regulatory cues that determine its function. To address this question we undertook a comprehensive characterization of INO80-C in human cells to identify putative relationships between chromatin signals and INO80-C function.

**Figure 1:**
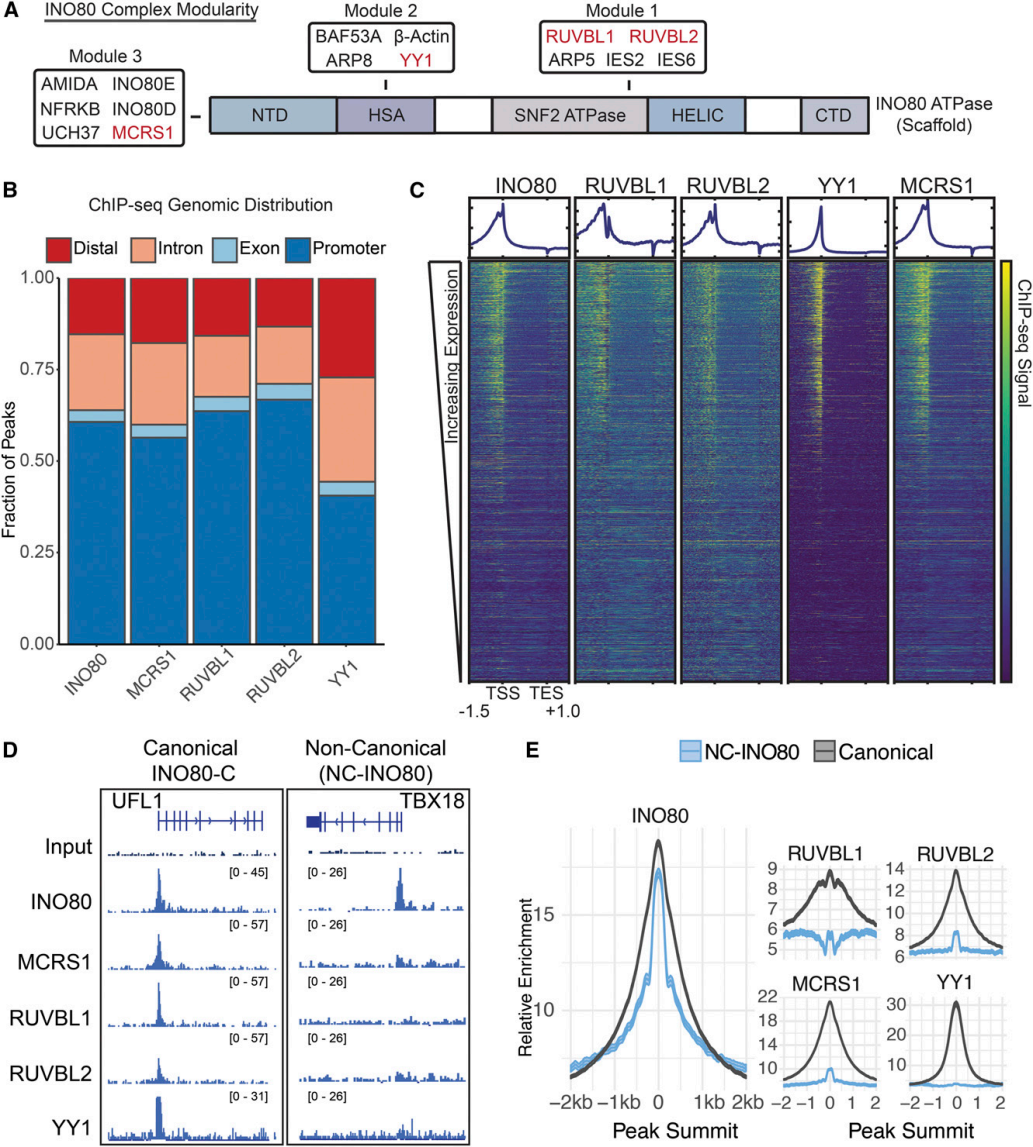
Genomic Occupancy of INO80-C Members Reveals Two Distinct Types of Sites. A. Schematic of INO80 protein and locations of binding modules, proteins mapped by ChIP-seq are highlighted in red. B. Genomic distribution of INO80-C members. C. ChIP-seq signal of INO80-C subunits aligned relative to gene units (-1.5kb upstream to +1kb downstream). Rows are ordered based on expression of all expressed genes in HepG2 cells (n = 17157). D. ChIP-seq signal example of Canonical INO80 and NC-INO80 sites. E. Metagene plots of signal for INO80 and 4 others mapped INO80-C subunits aligned to midpoint of NC-INO80 and Canonical INO80 sites (n = 4949 NC-INO80, n = 18716 Canonical).

## Materials and Methods

### Cell Culture

HepG2 cells were purchased from the ATCC and grown in DMEM (Gibco, Life Technologies) supplemented with 10% Fetal Bovine Serum and penicillin/streptomycin (100 units/mL).

### Antibodies

BAF53A: LifeSpan Biosciences LS-C196606 (Western). EZH2: Millipore 07-400 (ChIP, IP, Western). INO80: Abcam ab105451 (ChIP, IP, Western). MCRS1: Proteintech 11362-1-AP (ChIP). RUVBL1: Bethyl A304-716A (ChIP) and Abcam ab51500 (Western). RUVBL2: Bethyl A302-537A (ChIP) and Abcam ab89942 (Western). SUZ12: Cell Signaling 3737S (Western). YY1: Santa Cruz sc-1703 (Western).

### Chromatin Immunoprecipitation

#### Fixation:

HepG2 cells were fixed using 0.3% formaldehyde in 1X PBS for 30 min at 4°. Fixative was quenched with 1/10 volume of 2M glycine for 5 min at room temperature. Cells were pelleted at 2000 rpm for 10 min at 4°, then washed 3 times by resuspension in 50mLs of ice cold 1X PBS and centrifugation at 2000 rpm for 10 min at 4°. Cells were aliquoted at 20 million cells and snap frozen using liquid nitrogen.

#### MNase ChIP:

ChIP was performed as previously described (Raab *et al.* 2015). Briefly, prior to beginning the ChIP, we washed Dynal Protein A beads 3 times with PBS + 0.5% BSA at 4°. We resuspended beads in 400μL of 1X + 0.5% BSA, then added 4-10ug of antibody and incubated overnight at 4°. The following day, we thawed the fixed cells for 30 min on ice. We then resuspended each pellet in 1mL swelling buffer (25mM HEPES + 1.5mM MgCl2 + 10mM KCl + 0.1% Igepal). We added 10mM PMSF and Proteinase Inhibitor Cocktail, and pooled the samples. The pooled samples were inverted for 10 min at 4° then dounced 20 strokes. We pelleted nuclei at 2000 rpm for 7 min at 4°. Nuclei were resuspended in 5mL Sucrose Buffer A (0.32M Sucrose, 15mM Hepes pH 7.9, 60mM KCl, 2mM EDTA, 0.5mM EGTA) + PMSF and Proteinase Inhibitor Cocktail. Then 5mL Sucrose Buffer B (30% Sucrose, 15mM Hepes pH 7.9, 2mM EDTA, 0.5mM EGTA) was gently added. We centrifuged the sucrose solution at 3000 rpm for 10 min at 4°, then washed the pellet in 10mL MNase Digestion Buffer (15mM Hepes pH 7.9, 60mM KCl, 15mM NaCl, 0.32M Sucrose) at 2000 rpm for 7 min at 4°. The pellet was resuspended in 1mL MNase Digestion Buffer per 4e7 cells + 3.3μL 1M CaCl2 per mL + PMSF and Proteinase Inhibitor Cocktail, then incubated for 5 min at 37° to warm. We added MNase at 0.5μL/1e7 cells and incubated for 15 min at 37°. Following digestion, the MNase was chelated using 1/10 volume 0.5M EDTA on ice for 5 min. We added 1 volume of 2X IP Buffer (20mM TrisCl pH 8, 200mM NaCl, 1mM EDTA, 0.5mM EGTA), then passed the sample for a 20G needle 5 times and a 25G needle 5 times. The sample was split into microcentrifuge tubes and centrifuged at 13,000 rpm for 15 min at 4° to remove debri. We pooled the supernatants and placed on ice for 1 hr. The pellets were resuspended in 1mL 1X IP Buffer (10mM Tris-HCl pH 8, 100mM NaCl, 1mM EDTA, 0.5mM EGTA) and inverted for 1 hr at 4°. Then we centrifuged the sample and mixed the supernatant with S1. We added 1% Triton-X 100 to the chromatin. The sample was split evenly to the prepared beads, after they were washed twice with 1X PBS + 0.5% BSA and twice with 1X IP Buffer. We saved 10% of the sample for input and incubated the IPs overnight at 4°. The following day we washed the beads 10 times with Agilent RIPA wash by adding 1mL wash buffer and inverting for 4 min at 4°. Beads were subsequently washed one time with 1X TE + 50mM NaCl before elution at 65° with agitation using 100μL 1% SDS + 100mM NaHCO3 made freshly. We removed the supernatant and treated with 5μL of 5M NaCl overnight to reverse the crosslinks. We cleared RNAs and proteins by adding 3μL RNaseA for 45 min at 37C and then Proteinase K for 2 hr at 56°. Precipitates were purified using the Zymo Clean and Concentrator ChIP Kit and quantified using qubit before library preparation.

### Immunoprecipitation

#### Preparation of Nuclear Lysates:

Cells were washed with PBS, then centrifuged for at 1300 rpm for 10 min at 4°. Cells were washed with 20 packed cell volumes with hypotonic cell lysis buffer (10mM HEPES-KOH pH 7.9, 1.5mM MgCl2, 10mM KCl, 0.5mM DTT) plus protease inhibitors and placed on ice for 10 min to swell. Cells were then centrifuged at 1300 rpm for 10 min at 4°. Cells were dounced with 2 packed cell volumes of hypotonic cell lysis buffer. Nuclei were pelleted at 1300 rpm for 10min at 4°. Nuclei were washed with 10 packed cell volumes with hypotonic cell lysis buffer and centrifuged at 5000 rpm for 10 min. Extractions were performed with 60μL of nuclear lysis buffer (20mM HEPES-KOH pH 7.9, 25% glycerol, 420 mM KCl, 1.5mM MgCl2, 0.2mM EDTA, 0.5mM and protease inhibitors) per 100μL cell pellet. Lysates were clarified at 14,000 rpm for 10 min at 4° between extractions. Lysates were diluted with storage buffer (20mM HEPES-KOH pH 7.9, 20% glycerol, 0.2mM EDTA, 0.2mM DTT) to bring final KCl concentration to 150mM.

#### Immunoaffinity:

Prior to beginning the IP, we washed Dynal Protein A beads 3 times with PBS + 0.5% BSA at 4°. We resuspended beads in 400μL of 1X + 0.5% BSA, then added 5ug of antibody and incubated overnight at 4°. The following day, we thawed the nuclear lysates on ice. Lysates were added to antibody-conjugated beads and incubated overnight. Beads were washed 7 times for 5 min each, in this order: 2X with IP Buffer (20mM HEPES-KOH pH 7.9, 0.15M KCL, 10% glycerol, 0.2mM EDTA pH 8.0, 0.1% Tween-20, 0.5mM DTT, and protease inhibitors), 3X with Wash Buffer (20mM HEPES-KOH pH 7.9, 0.1M KCL, 10% glycerol, 0.2mM EDTA pH 8.0, 0.1% Tween-20, 0.5mM DTT, and protease inhibitors), and 2X Final Buffer (20mM HEPES-KOH, pH 7.9, 60mM KCl, 10% glycerol, 0.5mM DTT, and protease inhibitors). Proteins were eluted from beads using 2X Laemmli buffer with 1:10 DTT for 10 min at 95C. Beads were magnetized and supernatants stored at -20°.

### Glycerol Gradient Sedimentation

Density sedimentation was performed as previously described (Kadoch and Crabtree 2013). Briefly, 500μg of HepG2 nuclear lysates were suspended in 0% glycerol HEMG buffer (25mM HEPES pH 7.9, 0.1mM EDTA, 12.5mM MgCl2, 100mM KCL, DTT and PMSF), loaded into a glycerol gradient (10mL 10–30% glycerol in HEMG), and centrifuged at 40K RPM for 16 hr at 4°. 500μL fractions were collected, suspended in 2X Laemmli buffer with 1:10 DTT, and boiled for 5 min at 95° prior to western blot analyses.

### ChIP-seq analysis

#### Mapping:

Reads were aligned to hg19 using bowtie2 (Langmead and Salzberg 2012) using the–sensitive parameters, and duplicates were removed using samtools (Li *et al.* 2009). For visualization bigwig tracks were generated using Deeptools (version 2.2.2) bamCoverage tool with a binsize of 10bp and extending fragments to the approximate nucleosome size of 150bp. Tracks can be visualized using IGV (Robinson *et al.* 2011) and bw files are available in GEO Accession number GSE97411.

#### Peak Calling:

Peaks were called using Macs2 (version 2.1.0 (Zhang *et al.* 2008)) using the narrowpeak mode using the following parameters. Qval = 0.001–keep-dup-all–shift 37–nomodel–extsize 147. Additionally, we filtered the peaks against the ENCODE blacklist regions and further recursively merged any peaks within 500bp of the nearest peak.

#### Definition of Non-Canonical sites:

We defined Canonical INO80 and Non-Canonical INO80 sites using a simple overlap approach. We used the filtered and merged peak files generated above to define INO80 bound regions that did not have any overlap with any of the other INO80-C subunit sites.

#### Comparison With ENCODE Data:

Raw fastq files for YY1 were downloaded from the ENCODE project and processed as with other INO80-C subunits. H3K27ac, H3K27me3, and EZH2 data bam alignment files were downloaded and used to generate bigwig files as above. Coverage at regions of interest were generated using Deeptools (version 2.2.4 (Ramírez *et al.* 2016) with the computeMatrix function using a binsize of 10bp using the bigwig files generated above. These were further processed using the heatmapper module of deeptools or using ggplot2 in R (version 3.2.2).

### RNA-seq analysis

For gene expression analysis, we used our previous RNA-seq data in HepG2 cells (Raab *et al.* 2015). Expression analysis was carried out on single end 50bp reads. Gene expression levels were quantitated using kallisto (Bray *et al.* 2016). These data were converted to counts and summarized per gene using tximport (Soneson *et al.* 2015). When comparing genes associated with the two classes, we assigned genes to the nearest peak.

### Data Availability

All raw and processed data are deposited under accession number GSE97413.

ChIP-seq data are deposited under series GSE97411

Processed peak calls for ChIP can be found in Table S1.

## Results

### Identification of a Distinct INO80 Binding Class Devoid of Canonical Subunits

To investigate the factors that influence INO80-C function throughout the genome, we performed ChIP-seq for 4 subunits of INO80-C in the liver cancer cell line HepG2. We obtained data for a fifth subunit in HepG2, YY1, from ENCODE ((ENCODE Project Consortium 2012) Accession ENCFF000PSE, ENCFF000PSD). Together, the five subunits we mapped are representative of the three modules that form INO80-C (Figure 1A) (Chen *et al.* 2011). Our ChIP-seq experiments showed that human INO80-C subunits localized to genomic elements at a similar ratio to one another, consistent with a unified function (Figure 1B). The majority of binding sites were found proximal to the promoter, with a large fraction of peaks (∼40%) associated with distal or intronic regions. YY1 bound genomic elements at a moderately different proportion than INO80, RUVBL1, RUVBL2, and MCRS1, consistent with its known promiscuity as a DNA-binding regulatory partner for several other complexes (Gordon *et al.* 2006). Additionally, the INO80-C subunits were markedly enriched around transcription start sites (TSS) of highly expressed genes (Figure 1C). Interestingly, the subunits were not enriched at transcription end sites (TES) (Figure 1C), despite evidence that INO80-C binds both TSSs and TESs in yeast (Xue *et al.* 2015, 2017). This observation suggests that nucleosome depletion near the transcription end site does not depend on INO80-C in human cells (Fan *et al.* 2010). Such an organism-specific distinction is consistent with organismal differences in genome organization and the identification of mammalian specific INO80-C subunits that are not present in other eukaryotes (Jin *et al.* 2005).

We were surprised to find that 22% of INO80 ATPase peaks did not overlap RUVBL1, RUVBL2, MCRS1, or YY1 (Figure 1D, E). We separated INO80 ChIP-seq peaks into two categories to analyze this unexpected group further. The first category, named Canonical INO80, refers to sites where the INO80 ATPase displayed co-occurrent peaks with any other protein we mapped (RUVBL1, RUVBL2, MCRS1, and YY1). The second category, named Non-Canonical INO80 (NC-INO80), contains only the INO80 peaks that displayed no overlap with peaks of other INO80-C subunits. To determine the genome-wide enrichment of INO80-C subunits in the two categories, we measured signal for INO80, RUVBL1, RUVBL2, MCRS1, and YY1 in both (Figure 1E). INO80 itself displayed high signal in both categories (Figure 1E) indicating that the two groups are robust INO80 targets. Although the other subunits exhibited high signal at Canonical INO80 sites genome-wide as expected, they showed very low ChIP-seq signal at NC-INO80 sites (Figure 1E). The two categories of INO80 targeting also exhibited different widths for INO80 enrichment at the peak summit (Fig. S1A). Given the role of INO80 as a scaffold for its biochemical partners, we hypothesized that the NC-INO80 sites represented a meaningful unidentified class of INO80 targeting.

### NC-INO80 Class Harbors Heterochromatin Signature

**Figure 2:**
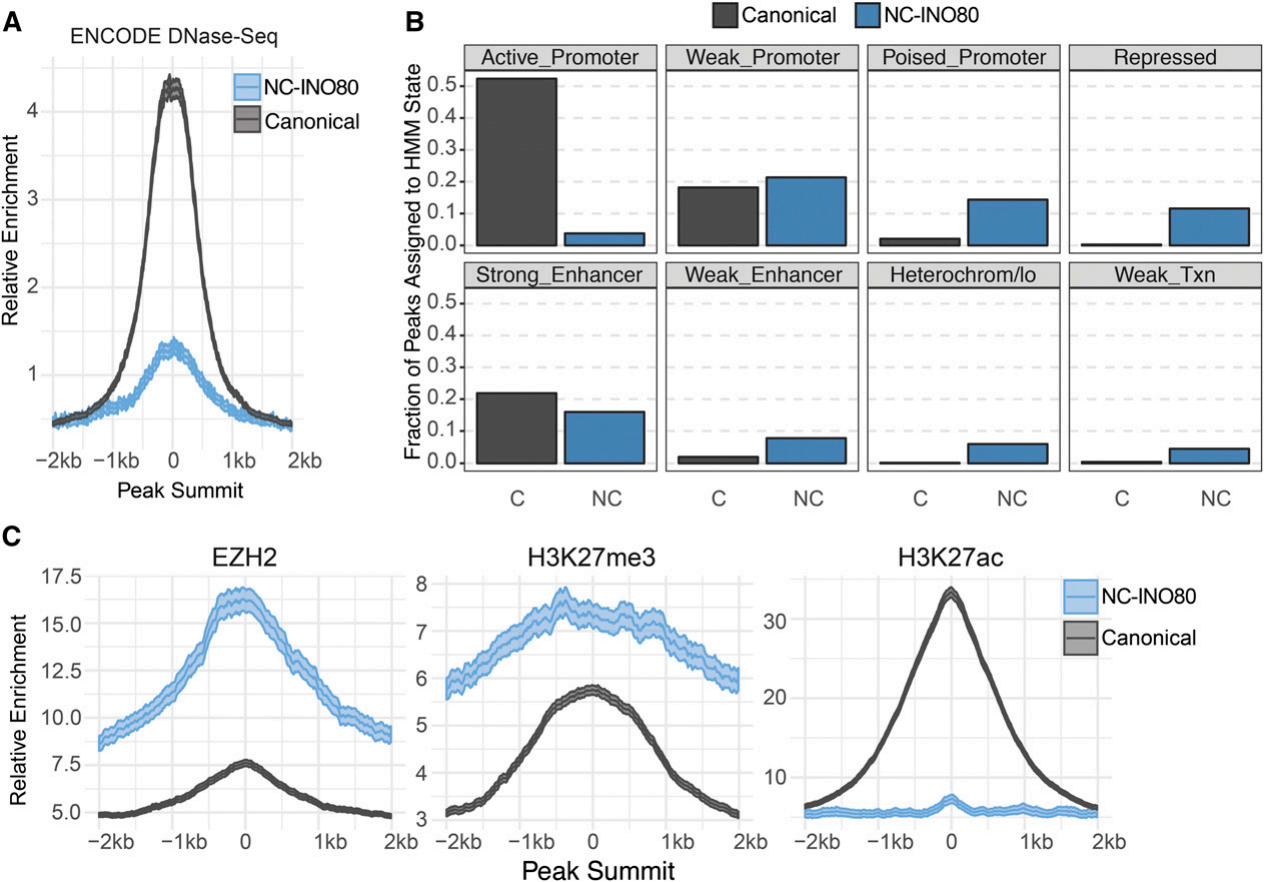
Characterization of Chromatin Features at Canonical and Non-Canonical INO80 Sites. A. DNase signal at each class of peaks generated using ENCODE Data (Accession #ENCSR149XIL). B. Fraction of INO80 peaks from each class that are localized to different types of ChromHMM states (Ernst *et al.* 2011). C. Metaplots centered on Canonical INO80 and NC-INO80 sites for EZH2, H3K27me3, and H3K27ac. Data were generated by ENCODE (Accessions ENCSR000ARI, ENCSR000AOL, ENCSR000AMO).

Biochemical evidence suggests the remodeling activity of INO80-C is most efficient when its subunit modules are intact. Therefore we measured chromatin accessibility at Canonical INO80 and NC-INO80 sites using ENCODE DNase-seq data in HepG2. Canonical INO80 sites displayed high signal by DNase-seq (Figure 2A). Because INO80-C binding is associated with highly expressed genes (Figure 1), these data support a model where INO80-C mobilizes nucleosomes to facilitate chromatin accessibility. In contrast, DNase-seq signal at NC-INO80 sites was low (Figure 2A) suggesting INO80 is not able to facilitate chromatin accessibility at these sites.

To identify distinguishing chromatin features that influence INO80 targeting, we intersected our ChIP-seq datasets with chromatin state predictions from ENCODE in HepG2 (ChromHMM (Ernst *et al.* 2011)). We found that Canonical INO80 and NC-INO80 targets were enriched for distinct chromatin states (Figure 2B). More than 70% of Canonical INO80 targets overlapped chromatin states representing highly active chromatin, including states associated with active promoters and strong enhancers. Alternatively, the majority of NC-INO80 targets (65%) were co-localized with repressive chromatin states. Only a very small fraction of NC-INO80 peaks resembled active promoters. Approximately 20% of both INO80 classes were enriched for states associated with weak promoters and strong enhancers. In addition, we analyzed ENCODE ChIP-seq datasets in HepG2 to investigate specific co-regulatory candidates that may influence INO80 function at the two classes. Notably, the EZH2 methyltransferase from the Polycomb Repressive Complex 2 (PRC2) and the primary post-translational modification it deposits on lysine 27 of histone 3 (H3K27me3) were highly enriched at NC-INO80 sites, whereas signal for EZH2 and H3K27me3 were markedly lower at Canonical INO80 sites (Figure 2C). Moreover, the opposing modification on lysine 27 of histone 3 (H3K27ac) was absent from NC-INO80 sites but highly enriched at the Canonical INO80 class (Figure 2C). These data suggest Canonical INO80 and NC-INO80 targets represent functionally different regulatory elements. While Canonical INO80 interacts with active genomic elements and co-localizes with H3K27ac, NC-INO80 is strongly enriched for heterochromatin features including PRC2 and H3K27me3.

**Figure 3:**
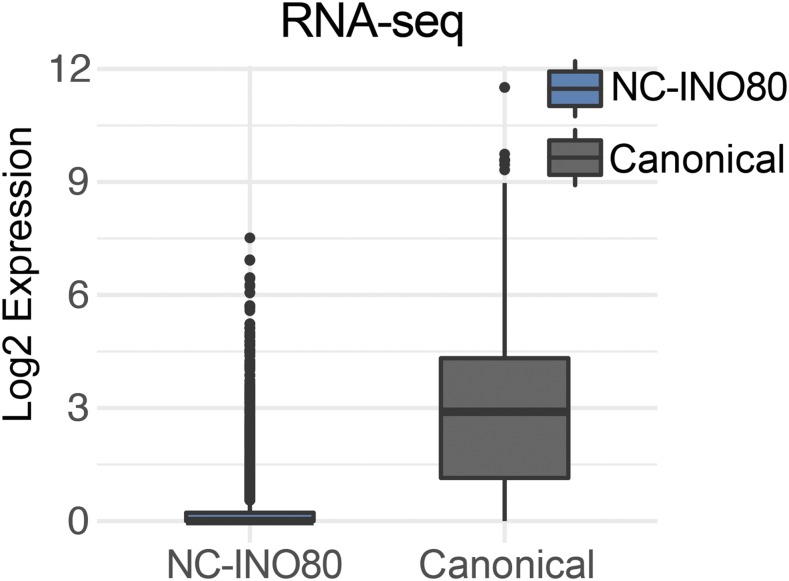
Expression of Genes near INO80 Targets Correlates with Local Chromatin State A. Gene expression in HepG2 of genes assigned by linear distance to INO80 peaks. Genes with assignments to peaks of both Canonical INO80 and NC-INO80 peaks were excluded.

Additionally, we used our previous RNA-seq data in HepG2 cells (Raab *et al.* 2015) to determine the expression level of genes near Canonical INO80 and NC-INO80 sites. We assigned genes to either Canonical INO80 or NC-INO80 based on the nearest INO80 ChIP-seq peak. Genes targeted by NC-INO80 were expressed at dramatically lower levels than genes targeted by Canonical INO80 (Figure 3A). These data correlate with the repressive chromatin signature at NC-INO80 targets and implicate each class with its expected regulatory output based on local chromatin features.

### Biochemical Evidence of INO80-C Crosstalk With Histone Modifying Enzymes

**Figure 4:**
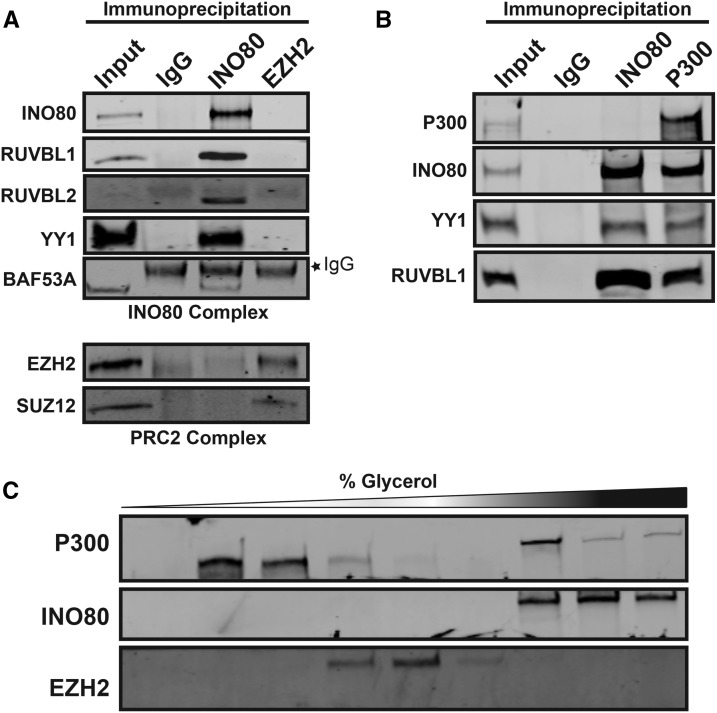
INO80 Interacts with the Histone Acetyltransferase P300. A, B. Immunoprecipitation for INO80 and EZH2 followed by western blotting for members of each complex. C. Western blots performed on fractions of glycerol gradient sedimentation in HepG2 cells.

We next performed a series of biochemical experiments to determine if INO80-C interacted with histone modifiers of the chromatin landscape from Canonical INO80 and NC-INO80 classes. First, we performed low-stringency co-immunoprecipitations to determine if INO80-C members interacted with PRC2. Although we detected robust interactions between members of the same complex, we did not detect any interaction between the INO80 ATPase and EZH2, or other members from opposite complexes (Figure 4A).

Surprisingly, we observed interactions between members of INO80-C and P300 by co-immunoprecipitation under the same conditions (Figure 4B). P300 is a histone acetyltransferase that deposits acetylation on H3K27 and is known to be antagonized by H3K27me3 from PRC2 (Pasini *et al.* 2010). Interestingly, although P300 immunoprecipitation yielded the INO80 ATPase and subunits of INO80-C, we did not detect reciprocal interactions between P300 and the INO80 ATPase so we utilized an orthologous approach to validate the interaction. We performed glycerol gradient sedimentation to determine if P300 and the INO80 ATPase migrated in overlapping fractions, which would suggest they exist in a similarly-sized protein complex and indicates a physical interaction between them likely occurs. Indeed, P300 and the INO80 ATPase displayed overlap, distinct from EZH2 (Figure 4C). Importantly, P300 displayed a bimodal distribution where P300 was abundant in low glycerol concentrations suggesting an INO80-independent P300 likely exists. These data implicate INO80-C remodeling capabilities with the acetyltransferase activity of P300 but not necessarily vice versa.

## Discussion

**Figure 5:**
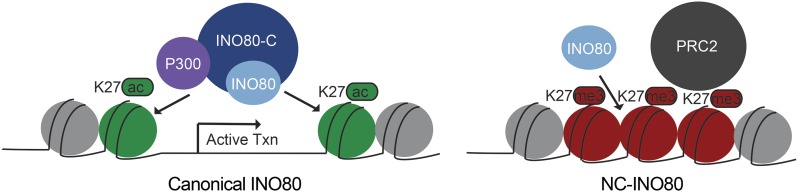
Two Distinct Classes of INO80-C Binding Genome-wide. A. Model illustrating the distinguishing characteristics of Canonical INO80 and NC-INO80 binding in HepG2 cells.

Our data suggest that two remarkably distinct classes of INO80-C targeting exist in human cells (Figure 5). A class we named Canonical INO80 contained a histone modification signature consistent with active chromatin states (DNase sensitivity, H3K27ac) and was occupied by all the INO80-C subunits we mapped. The second class was marked by repressive chromatin features (DNase insensitivity, EZH2 and H3K27me3) and surprisingly lacked the INO80-C accessory subunits RUVBL1, RUVBL2, MCRS1, and YY1. We named this second class Non-Canonical INO80 (NC-INO80). Genes near Canonical INO80 exhibited high levels of expression, whereas genes near NC-INO80 were lowly expressed. We identified that INO80-C physically interacted with P300 under endogenous conditions, suggesting INO80-C and P300 coordinate chromatin accessibility jointly at Canonical INO80 sites. We did not detect interactions between INO80-C and PRC2 indicating that either these two complexes do not physically interact at NC-INO80 sites or that they may interact through non-physical means (antagonistic, sequential, mutually exclusive) that our genomics and biochemical approaches are unable to resolve. Given the stark differences in epigenomic signatures at the two classes of targets, these results suggest that local chromatin landscape is important to INO80-C targeting and function.

Our discovery that NC-INO80 correlates with inaccessible chromatin suggests that the INO80 ATPase binds widely across the genome, and that the appropriate chromatin context may determine the ability of INO80-C to assemble and remodel chromatin. This is consistent with a scaffolding function for the INO80 protein and its requirement for interaction with its accessory subunits to mobilize nucleosomes (Jónsson *et al.* 2004; Chen *et al.* 2011, 2013; Watanabe *et al.* 2015; Yao *et al.* 2016; Willhoft *et al.* 2016).

Zhou *et al.* recently proposed that *in vivo* assembly of INO80-C occurs in a stepwise fashion, which may explain the differences in subunit localization and chromatin features at Canonical INO80 and NC-INO80 targets (Zhou *et al.* 2017). In addition to their identification of a robust heterododecameric assembly of RUVBL1 and RUVBL2 bound to the INO80 ATPase, the authors found that RUVBL1 and RUVBL2 form a heterohexamer independently from INO80-C in yeast. Evidence suggests the recognition of the INO80 ATPase by RUVBL1-RUVBL2 and co-stimulation by ATP hydrolysis leads RUVBL1-RUVBL2 to facilitate complete INO80-C assembly and activity (Jónsson et al. 2004; Chen, Conaway, and Conaway 2013; Zhou et al. 2017). The authors suggested that perhaps after the remodeling function of INO80-C is completed, RUVBL1-RUVBL2 may be subsequently ejected from INO80-C, leading to disassembly of the complex, and recycling of RUVBL1-RUVBL2 as they await the appropriate signals to initiate and complete INO80-C assembly again. 

Aside from a model for stepwise assembly of INO80-C *in vivo*, one alternative explanation could be that INO80-C forms an array of complexes and subcomplexes throughout the genome, with each comprised of distinct sets of proteins that facilitate specific functions. Similar remodeling complexes to INO80-C, such as SWI/SNF, have been shown to exhibit striking compositional plasticity (Hodges *et al.* 2016). Genomics studies have demonstrated that variable SWI/SNF subunits exert discrete functions at loci that are differentially occupied (Euskirchen *et al.* 2011; Raab *et al.* 2015, 2017). For SWI/SNF, it has been proposed that this variability indicates that cells may contain dozens or even hundreds of SWI/SNF compositions (Kadoch and Crabtree 2015). For INO80-C, there have been far fewer instances of heterogeneity described in the literature to date. Our data suggest that at least two types of INO80-C target chromatin in human cells. While there may be subsets of complexes throughout the genome that our classification strategy does not address or that variability in antibody efficiency prevents us from detecting, it seems possible INO80-C is not quite as variable as SWI/SNF and instead contains a few specific subtypes and formations.

For instance, Xue *et al.* recently demonstrated that the INO80 ATPase forms a complex with transcriptional modifiers MOT1 and NC2, termed MINC (Xue *et al.* 2017). MINC suppressed transcription at its targets in yeast and murine embryonic stem cells. MOT1 and NC2 had been shown to jointly suppress transcription by inhibiting the function of the transcriptional activator TBP (van Werven *et al.* 2008; Koster *et al.* 2014) suggesting MINC antagonizes gene activation. Importantly, a subset of MINC targets correlated with factors from the Polycomb Repressive Complex 1 and exhibited low levels of TBP. These sites closely resemble the NC-INO80 targets we describe.

Interestingly, MINC also suppressed transcription at a set of targets that harbored active chromatin features (Xue *et al.* 2017). The localization of MINC to both active and repressive chromatin targets is consistent with our observations, although Canonical INO80 and NC-INO80 classes contain distinct INO80-C compositions. Perhaps MINC is a specialized variant of INO80-C, as other groups have demonstrated that INO80-C directs gene activation, as well as gene repression, in several contexts (Cai *et al.* 2007; Wang *et al.* 2014; Yao *et al.* 2016; Zhou *et al.* 2016; Gowans *et al.* 2018). In total, results from our group and others point to an impressive level of complexity for INO80-C in its targeting, molecular assembly, and functional output that is only beginning to be appreciated.

## Supplementary Material

Supplemental Material is available online at www.g3journal.org/lookup/suppl/doi:10.1534/g3.117.300504/-/DC1.
