# Supplementary figures and images for: Identification of Two Distinct Classes of the Human INO80 Complex Genome-Wide
Source: G3 (Bethesda). 2018 Mar 6;8(4):1095–102. doi: 10.1534/g3.117.300504 (PMC5873900; doi:10.1534/g3.117.300504)

**A**

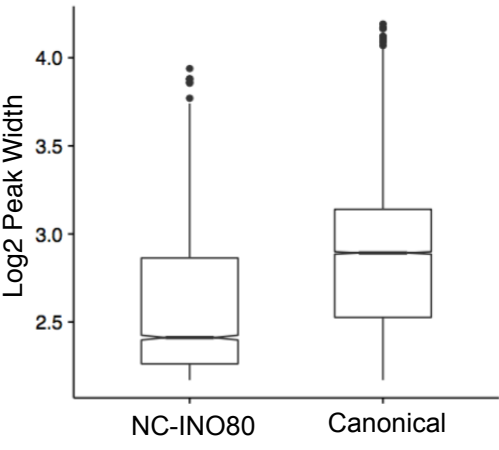

Supplement: Supplementary file 1 [file 1095FigureS1.pdf]
